# Ingenious Action of *Vibrio cholerae* Neuraminidase Recruiting Additional GM1 Cholera Toxin Receptors for Primary Human Colon Epithelial Cells

**DOI:** 10.3390/microorganisms10061255

**Published:** 2022-06-20

**Authors:** Johanna Detzner, Charlotte Püttmann, Gottfried Pohlentz, Johannes Müthing

**Affiliations:** Institute for Hygiene, University of Münster, 48149 Munster, Germany; johanna.detzner@ukmuenster.de (J.D.); lotte02021996@web.de (C.P.); mgmj.pohlentz@gmx.de (G.P.)

**Keywords:** colon epithelial cells, choleragenoid, cholera toxin, gangliosides, GM1, GD1a, neuraminidase, pHCoEpiCs, *Vibrio cholerae*

## Abstract

For five decades it has been known that the pentamer of B subunits (choleragenoid) of the cholera toxin (CT) of *Vibrio cholerae* binds with high preference to the ganglioside GM1 (II^3^Neu5Ac-Gg4Cer). However, the exact structures of CT-binding GM1 lipoforms of primary human colon epithelial cells (pHCoEpiCs) have not yet been described in detail. The same holds true for generating further GM1 receptor molecules from higher sialylated gangliosides with a GM1 core through the neuraminidase of *V. cholerae*. To avoid the artificial incorporation of exogenous gangliosides from animal serum harboring GM1 and higher sialylated ganglio-series gangliosides, pHCoEpiCs were cultured in serum-free medium. Thin-layer chromatography overlay binding assays using a choleragenoid combined with electrospray ionization mass spectrometry revealed GM1 lipoforms with sphingosine (d18:1) as the sole sphingoid base linked to C14:0, C16:0, C18:0 or C20:0 fatty acyl chains forming the ceramide (Cer) moieties of the main choleragenoid-binding GM1 species. Desialylation of GD1a (IV^3^Neu5Ac,II^3^Neu5Ac-Gg4Cer) and GT1b (IV^3^Neu5Ac,II^3^(Neu5Ac)_2_-Gg4Cer) of pHCoEpiCs by *V. cholerae* neuraminidase was observed. GD1a-derived GM1 species with stable sphingosine (d18:1) and saturated fatty acyl chains varying in chain length from C16:0 up to C22:0 could be identified, indicating the ingenious interplay between CT and the neuraminidase of *V. cholerae* recruiting additional GM1 receptors of pHCoEpiCs.

## 1. Introduction

Cholera is caused by the human pathogen *Vibrio cholerae*, which is the causative agent of severe dehydrating diarrheal disease with epidemic and pandemic potential of the O1 and O139 serotypes [1,2]. Severe cholera is distinct from other diarrheal illnesses, with pronounced diarrheal purging that requires aggressive fluid replacement and/or antibiotic treatment [2,3]. Infection is initiated by pathogen colonization of the small intestine after passage of the acid barrier in the stomach and survival in the intestinal lumen facing bile and antimicrobial peptides. Among the more than 200 proteins functionally linked to the virulence-associated genes of *V. cholerae*, the eponymous toxin is the main virulence factor in the development of cholera, being responsible for the lethal symptoms of the disease [3,4]. Cholera toxin (CT, choleragen) is a heterohexameric AB_5_ toxin composed of an A subunit (CTA, 26 kDa) and a B pentamer (CTB, choleragenoid) consisting of five identical 11.6 kDa sized B subunits [4,5,6,7]. CTA consists of two domains, A1 and A2, and the A1-chain is held by the tethering the A2-chain above the plane of the B pentamer. A1 and A2 are linked by an exposed loop harboring a proteolytic cleavage site and a disulfide bond that bridges the cleavage site fragment. The B pentamer binds to GM1 gangliosides exposed on the luminal surface of intestinal epithelial cells and directs the A subunit in a retrograde manner through the endosomes and the *trans*-Golgi network (TGN) to the endoplasmic reticulum (ER). Upon delivery to the cytosol, proteolytic cleavage of CTA and reduction of the disulfide bond generates the enzymatically active A1-fragment, which unfolds and enters the cytosol by hijacking components of the ER-associated degradation pathway for misfolded proteins [8,9,10] and crossing the ER limiting membrane by a process termed retro-translocation [11]. In the cytosol, the A1-chain rapidly refolds and activates the adenylate cyclase leading to increased intracellular cAMP and finally to impaired sodium uptake and increased chloride outflow, causing massive fluid secretion and fulminant watery diarrhea [3,4,5,12]. Interestingly, a fraction of cell surface-bound CT also moves across the cell to the basolateral plasma membrane by transcytosis, thus traversing the intestinal barrier [13].

**Figure 1:**
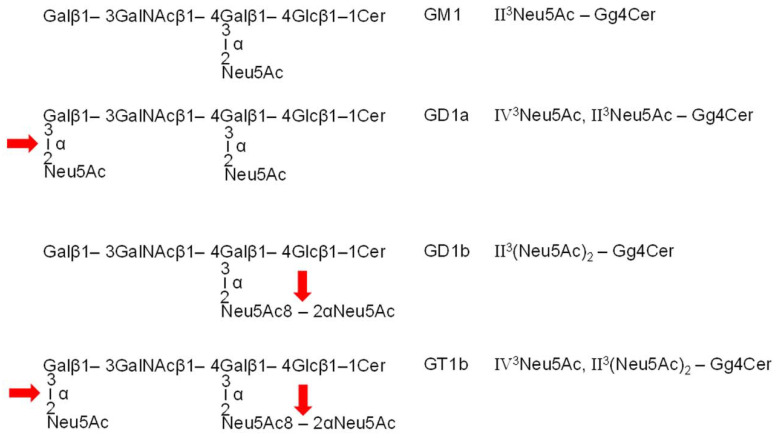
Structures of ganglio-series gangliosides GM1, GD1a, GD1b, and GT1b. The red arrows indicate the neuraminidase-sensitive positions of Neu5Ac terminally linked in α2-3-configuration to the distal galactose at position IV of GD1a and GT1b and the α2-8-linked outer Neu5Ac of the disialo-group of GD1b and GT1b at the proximal galactose at position II of the Gg4Cer core. For ganglioside nomenclature refer to the recommendations of the IUPAC-IUB Joint Commission on Biochemical Nomenclature [15] and the acronyms of brain gangliosides introduced by Svennerholm [17,18]. Gg4Cer, gangliotetraosylceramide; Neu5Ac, *N*-acetylneuraminic acid.

Ganglioside GM1, first structurally characterized by Kuhn and Wiegandt in 1963 [14], consists of a gangliotetraosylceramide (Gg4Cer) core with a Galβ1-3GalNAcβ1-4Galβ1-4Glcβ1-1Cer structure, which carries one α2-3-linked *N*-acetylneuraminic acid (Neu5Ac) molecule at the galactose in position II of the Gg4 tetrasaccharide (for structure see Figure 1). GM1 with a Galβ1-3GalNAcβ1-4(Neu5Acα2-3)Galβ1-4Glcβ1-1Cer structure is systematically termed II^3^Neu5Ac-Gg4Cer according to the nomenclature recommendations of the International Union of Pure and Applied Chemistry (IUPAC)-International Union of Biochemistry (IUB) [15]. For past studies and the future potential of GM1 ganglioside, refer to the review of Aureli et al. published in 2016 [16]. Further sialylation at the terminal galactose in position IV of GM1 monosialoganglioside results in the disialoganglioside IV^3^Neu5Ac,II^3^Neu5Ac-Gg4Cer, designated as GD1a, while the addition of an α2-8-linked Neu5Ac to the internal Neu5Ac of GM1 results in the isomeric disialoganglioside II^3^(Neu5Ac)_2_-Gg4Cer assigned as GD1b harboring the characteristic disialylated Neu5Acα2-8Neu5Ac-group (see Figure 1). An example of a further sialylated GM1-core ganglioside is the trisialoganglioside GT1b with the formula IV^3^Neu5Ac,II^3^(Neu5Ac)_2_-Gg4Cer (for structure see Figure 1). These four gangliosides have been known of for several decades, in addition to the neutral monohexosylceramide GalCer, as the dominant glycosphingolipids (GSLs) of the human brain [17,18]. This short excursion in composition and nomenclature of ganglio-series gangliosides is required in order to understand the complementary interaction of the receptor-binding choleragenoid (the B pentamer) of CT of *V. cholerae* and its intrinsic neuraminidase, which the pathogen releases together with the toxin into the environmental gut. As mentioned above, GM1 is the specific receptor of CT [19,20], and the neuraminidase of *V. cholerae* is capable of desialylating the mentioned higher sialylated gangliosides GD1a, GD1b, and GT1b to the basic GM1 structure, as shown in Figure 1, where the enzymatic cleavage sites are highlighted with red arrows. The Neu5Ac molecule linked to the internal galactose at position II of the Gg4Cer core is resistant to *V. cholerae* neuraminidase [14,21]. This feature enables *V. cholerae* to generate additional GM1 receptor molecules for its toxin through enzymatic conversion of the higher sialylated ganglio-series gangliosides with GM1 core structure to GM1.

Here we provide data on the exact structures of CT-binding GM1 gangliosides of primary human colon epithelial cells (pHCoEpiCs) and the recruitment of additional GM1 receptors for CT by *V. cholerae* neuraminidase using ganglioside preparations of pHCoEpiCs cultivated under serum-free conditions.

## 2. Materials and Methods

The materials used and the methods employed have been described in detail in previous work. Brief methodological descriptions are provided in the following sections together with the appropriate citations.

### 2.1. Cultivation of Primary Human Colon Epithelial Cells

Primary human colon epithelial cells (pHCoEpiCs) were purchased from ScienCell^TM^ (Carlsbad, CA, USA; Cat. No. 2950). The small letter “p” marks the source of the cells from the human colon in order to differentiate these “primary” cells from tumor-derived and genetically or otherwise immortalized colon epithelial cell lines. Immediately upon receipt, cells were sowed and propagated to an appropriate amount of cells, which served as a basis for the establishment of a master cell bank of one homogenous batch of pHCoEpiCs from passage number 4. For the experimental work, aliquots of the master bank were thawed and cultured in a humidified atmosphere (37 °C, 5% CO_2_) in special Colonic Epithelial Cell Medium (CoEpiCM; ScienCell^TM^, Cat. No. 2951). The primary colonic cells were cultured without any serum addition under serum-free conditions and without antibiotics. The modus operandi for cell cultivation and passages, microscopic cell control, recording of the cell morphology and data documentation has been previously described in standard work protocols [22,23,24].

### 2.2. Extraction of Lipids and Isolation of Gangliosides from Primary Human Colon Epithelial Cells

Lipid extraction of pHCoEpiCs was conducted in the same way as previously described to by us for primary human kidney and colon epithelial cells [22,23,24]. Lipids were extracted with methanol followed by the stepwise extraction with mixtures of chloroform and methanol with decreasing polarity using chloroform/methanol (1/2, *v*/*v*), chloroform/methanol (1/1, *v*/*v*) and chloroform/methanol (2/1, *v*/*v*). After solvent evaporation, alkali-labile glycerophospholipids and triglycerides were saponified using 1 M methanolic NaOH. Neutralization was done by the careful dropwise addition of 10 M HCl. The resulting NaCl and other small molecules were eliminated by dialysis. Water was then removed by lyophilization, and the dried sample was taken up in chloroform/methanol/water (30/60/8, *v*/*v*/*v*). Gangliosides were isolated by anion-exchange chromatography on a small column of DEAE-Sepharose CL-6B (GE Healthcare, Munich, Germany) as described [25]. Finally, the ganglioside fraction was dissolved in chloroform/methanol (2/1, *v/v*), transferred in a screw cap glass tube with a Teflon seal (Pyrex, Châteauroux, France) corresponding to 1 × 10^5^ cells/µL and stored at −20 °C until use.

### 2.3. Choleragenoid, Antibodies, Neuraminidases and Ganglioside Reference

The compounds used for the thin-layer chromatography (TLC) overlay assays with choleragenoid (B subunit of cholera toxin) have been described in detail in previous publications [26,27]. The cholera toxin B subunit was from Sigma-Aldrich (C-9903; Taufkirchen, Germany), goat anti-choleragenoid antiserum was from Calbiochem (no. 227040, lot 325992; Frankfurt, Germany) and secondary alkaline phosphatase (AP)-labeled rabbit anti-goat IgG antibody was sourced from Jackson ImmunoResearch (no. 305-055-003, lot 81754; West Grove, PA, USA). AP activity was detected with 5-bromo-4-chloro-3-indolyl phosphate *p*-toluidine salt (BCIP, Roth, Karlsruhe, Germany). *Vibrio cholerae* neuraminidase was from Sigma-Aldrich (N7885-1UN, batch #0000104129; Taufkirchen, Germany), *Arthrobacter ureafaciens* neuraminidase was from Boehringer (269611, lot 12375822-13; Mannheim, Germany) and *Clostridium perfringens* neuraminidase was from Sigma-Aldrich (N2876-6UN, lot SLCD5639; Taufkirchen, Germany). A reference mixture of human brain gangliosides (HBG) composed of GM1, GD1a, GD1b and GT1b, served as a positive control for the choleragenoid TLC overlay assays according to previous studies [26,27].

### 2.4. Thin-Layer Chromatography and Detection of GM1 Using Choleragenoid Combined with Neuraminidase Treatment

Glass-backed high-performance TLC plates (HPTLC plates, size 10 cm × 10 cm, 0.2 mm silica gel layer, cat. no. 1.05633.0001; Merck, Darmstadt, Germany) were used. Gangliosides were administered to the silica gel surface with a semi-automatic sample applicator (Linomat 5, CAMAG, Muttenz, Switzerland), separated in chloroform/methanol/water (120/85/20, *v*/*v*/*v*) supplemented with 2 mM CaCl_2_ and stained with 0.2% orcinol (*w*/*v*) in sulfuric acid/deionized water (3/1, *v*/*v*) [28]. For the TLC overlay assay with choleragenoid [26,27], the silica gel layer of the plates with separated gangliosides was fixed with polyisobutylmethacrylate (Plexigum P28, Röhm, Darmstadt, Germany). Impregnated plates were then incubated overnight at room temperature with 2.5 mU/mL of each neuraminidase in an appropriate buffer or in buffer alone (controls). The buffers used were: 0.05 M sodium acetate, 9 mM CaCl_2_, pH 5.5 for *V. cholerae* neuraminidase, 0.1 M sodium acetate, pH 4.8 for *A. ureafaciens* neuraminidase and 0.1 M sodium acetate, pH 5.4 for *C. perfringens* neuraminidase. Plates were then overlayed for 1 h with 250 ng/mL of choleragenoid in phosphate-buffered saline (PBS) containing 1% (*w*/*v*) bovine serum albumin (solution A). Goat anti-choleragenoid antiserum was applied as a 1:1000 dilution and AP-conjugated rabbit anti-goat IgG secondary antibody as 1:2000 dilution, each for 1 h and diluted in solution A [26,27]. BCIP was used with 0.05% (*w*/*v*) dissolved in glycine solution (pH 10.4) as the substrate for AP of the secondary antibody that generates an insoluble blue precipitate indicating ganglioside-bound choleragenoid on the silica gel layer of the HPTLC plate. Basics of the TLC overlay procedure have been published recently, providing all of the methodological details, especially exact descriptions of trickery practical handling, for example, of glycosphingolipid separation and silica gel fixation, exemplified with the Shiga toxin, which is an AB_5_ toxin comparable to the CT used in this study [28].

### 2.5. Structural Characterization of Gangliosides by Electrospray Ionization Mass Spectrometry

Nano electrospray ionization mass spectrometry (nanoESI MS) of gangliosides was performed using a SYNAPT G2-S mass spectrometer (Waters, Manchester, UK) equipped with a Z-spray source as previously described [22,23,24]. Gangliosides were extracted with methanol from silica gel that has been scraped from choleragenoid positive areas of the TLC plate, and extracts were directly applied to MS analysis. MS^1^ spectra were recorded in the negative ion mode with the following source settings: temperature 80 °C, capillary voltage 0.8 kV, sampling cone voltage 20 V, and offset voltage 50 V.

## 3. Results

Two replicates of serum-free cultivated pHCoEpiCs (R1 and R2) were produced using cells from early passages 4 and 5 as previously described for studies of Shiga toxins from pathogenic *Escherichia coli* bacteria [22,23,24]. The same conditions were applied for the choleragenoid receptor studies of in vitro propagated pHCoEpiCs shown in this study. Serum-free conditions were chosen, because GSLs from serum supplements can be taken up and incorporated in the plasma membrane of cultured cells. We therefore isolated ganglioside fractions from serum-free grown pHCoEpiCs and performed a biochemical and mass spectrometric analysis of choleragenoid-binding GM1 species of the cells. To this end, we first probed the neuraminidases of *V. cholerae*, *C. perfringens*, and *A. ureafaciens* for their capability to desialylate the disialogangliosides GD1a and GD1b and the trisialoganglioside GT1b to GM1 using a reference mixture of human brain gangliosides (HBG) known to contain the mentioned ganglio-series gangliosides (for structures see Figure 1). The neuraminidase of *V. cholerae* was then employed for probing ganglioside preparations of pHCoEpiCs for the presence of GM1 and higher sialylated gangliosides with a GM1 core, which could serve as “precursors” for generating further GM1 receptor molecules for CT of *V. cholerae* by enzymatic degradation of higher sialylated gangliosides to GM1. This is the first report that describes the various lipoforms of the CT receptor ganglioside GM1 and the characterization of the recruited additional GM1 receptor molecules of primary human colon epithelial cells with *V. cholerae* neuraminidase.

### 3.1. Action of Bacterial Neuraminidases on Reference Ganglio-Series Gangliosides with GM1-Core

Figure 1 shows the structures of the ganglio-series gangliosides GM1, GD1a, GD1b, and GT1b, which are common constituents of the animal and human brain [18]. The disialogangliosides GD1a and GD1b as well as the trisialoganglioside GT1b have a common GM1 core, which carries a Neu5Ac molecule at the proximal galactose at position II of the Gg4 oligosaccharide. This internal sialic acid is resistant towards enzymatic cleavage by bacterial neuraminidases. All the other binding positions of further Neu5Ac molecules of disialylated GD1a and GD1b as well as trisialylated GT1b are sensitive to neuraminidase, which converts these higher sialylated gangliosides to GM1. The neuraminidase cleavage sites are marked by red arrows in Figure 1, indicating as the result of enzymatic attack the cholera toxin receptor GM1 being resistant to further hydrolytic desialylation by the bacterial neuraminidases used in this study.

### 3.2. TLC Overlay Detection of Choleragenoid-Binding GM1 Using Reference Gangliosides from Human Brain

**Figure 2:**
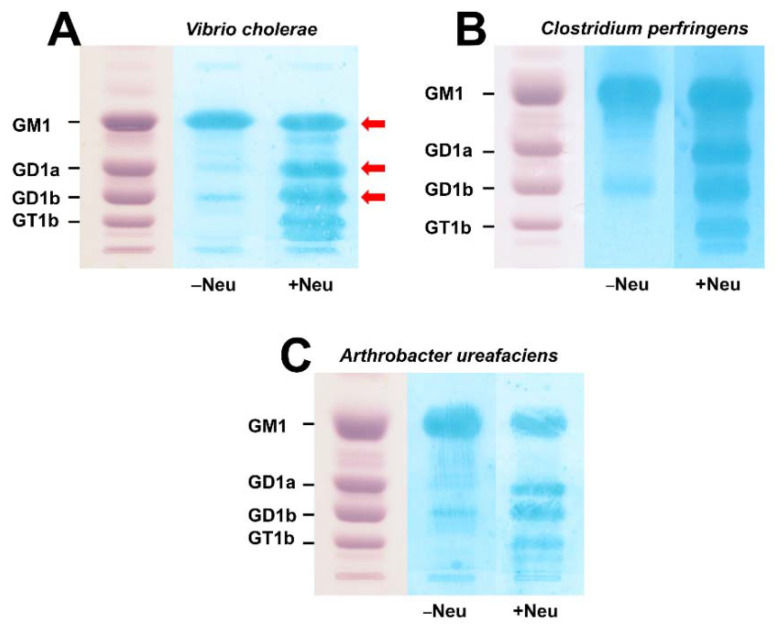
Detection of GM1 in a reference mixture of human brain gangliosides before and after treatment with the neuraminidase of *Vibrio cholerae* (**A**), *Clostridium perfringens* (**B**), and *Arthrobacter ureafaciens* (**C**). GM1 was detected without (−Neu) and after neuraminidase treatment (+Neu) by means of TLC overlay assays using choleragenoid (cholera toxin B subunit). Amounts of 15 µg of human brain gangliosides (HBG) were applied for the orcinol stains (left lanes of panel (**A**), (**B**), and (**C**)) and 1 µg for each overlay assay without (−Neu) and after neuraminidase treatment (+Neu). Structures of GM1, GD1a, GD1b and GT1b are shown in Figure 1. Mass spectrometric characterized gangliosides obtained from the silica gel extracts of TLC bands at the positions of GM1, GD1a and GD1b (see Figure 3), marked with red arrows, are listed in Table 1.

Figure 2 shows the proof of principle of choleragenoid-mediated overlay detection of GM1 in a TLC-separated mixture of human brain gangliosides (HBG) without (−Neu) and after treatment (+Neu) of the chromatograms with the neuraminidases of *V. cholerae* (Figure 2A), *C. perfringens* (Figure 2B) and *A. ureafaciens* (Figure 2C) prior to overlay analysis. The parallel orcinol-stained chromatograms of HBG in each panel of Figure 2 indicate the positions of GM1, GD1a, GD1b and GT1b on the TLC plate (for structures see Figure 1). Incubation in the presence of buffer without enzyme (−Neu) revealed strong binding of choleragenoid to GM1 accompanied by slight cross reaction with GD1b in the buffer controls. Incubation of the chromatograms with the three bacterial neuraminidases (+Neu) prior to exposure of the gangliosides to choleragenoid gave additional GM1-positive spots at the positions of GD1a, GD1b and GT1b. This result affirms the reliability of choleragenoid-mediated TLC overlay detection of primordial GM1 and further GM1 species derived from “precursor” disialogangliosides GD1a and GD1b and trisialoganglioside GT1b directly on the chromatogram after neuraminidase pretreatment at their chromatographic positions. The red arrows of the *V. cholerae* neuraminidase-treated lane (Figure 2A) indicate the positions from which the silica gel was scraped and ganglioside extracts were prepared for mass spectrometric analysis (see next paragraph).

### 3.3. Mass Spectrometric Characterization of Choleragenoid-Binding GM1 Lipoforms in Human Brain Gangliosides

**Figure 3:**
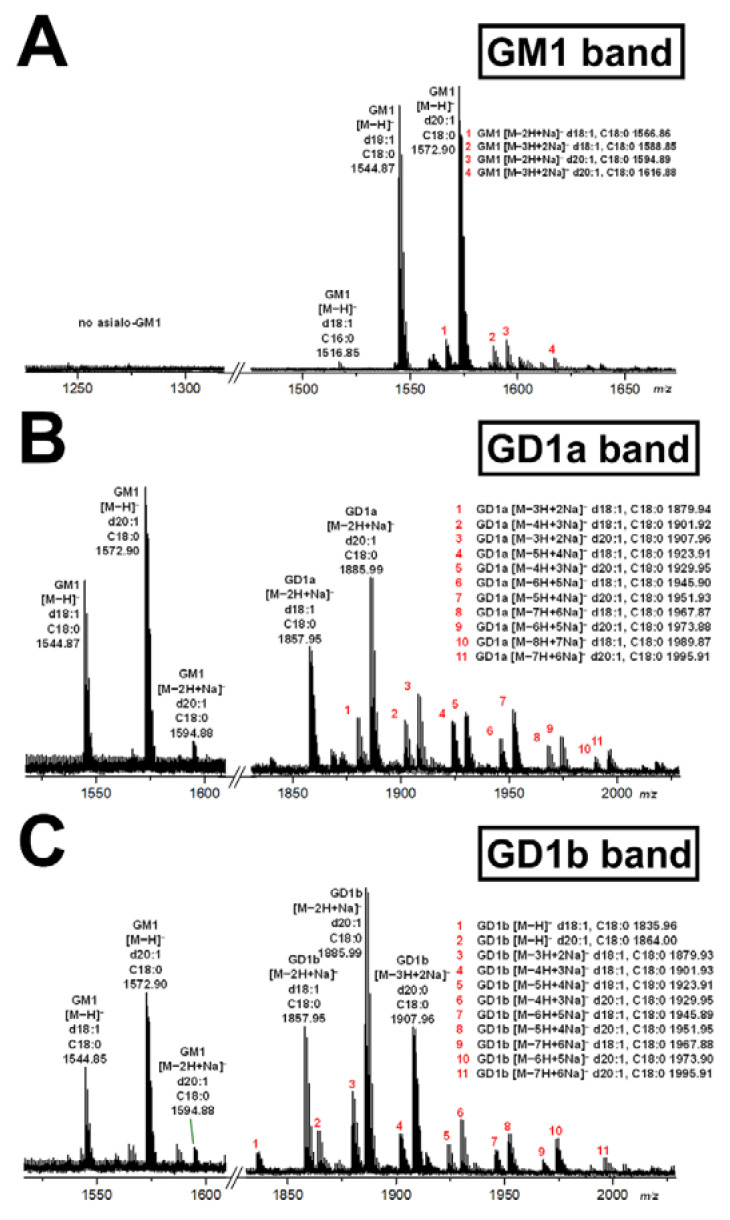
MS^1^ spectra of choleragenoid-detected GM1 species of reference human brain gangliosides at TLC positions of GM1 (**A**), GD1a (**B**) and GD1b (**C**) after treatment with neuraminidase of *Vibrio cholerae*. Separated gangliosides were extracted after *V. cholerae* neuraminidase treatment from scraped silica gel at positions of the TLC bands of GM1, GD1a and GT1b, which are marked by red arrows in Figure 2A. Spectra were recorded in the negative ion mode. The major signals of the deprotonated [M−H]^−^ species of GM1 and the deprotonated sodium adducts [M−2H+Na]^−^ of GD1a and the [M−2H+Na]^−^ and [M−3H+2Na]^−^ adducts of GD1b are listed in Table 1. Numbered minor species of GM1 (**A**) and the “precursor” disialogangliosides GD1a (**B**) and GD1b (**C**) are depicted on the right sides of the panels.

Structural characterization of extracted gangliosides obtained from scraped silica gel samples of the GM1, GD1a and GD1b bands of HBG (see Figure 2A) was performed by means of high-resolution electrospray ionization mass spectrometry (ESI-MS) and the achieved spectra are shown in Figure 3. The identified primordial GM1 lipoforms from the TLC-separated GM1 band were GM1 (d18:1, C18:0) and GM1 (d20:1, C18:0) at *m*/*z* 1544.87 and 1572.90, respectively, detected as deprotonated species [M−H]^−^ as shown in Figure 3A (for listing see Table 1). The MS^1^ spectrum indicated high abundance and approximately equal content of these two GM1 lipoforms accompanied by very minor counterparts appearing as deprotonated [M−2H+Na]^−^ and [M−3H+2Na]^−^ sodium adducts, which are indicated by red numbers and explained in the spectrum. The absence of asialo-GM1 (Gg4Cer) molecules, which would derive from GM1 (d18:1, C18:0) and GM1 (d20:1, C18:0) and would appear at *m*/*z* 1253.77 and 1281.80, respectively, underscored the resistance of internal Neu5Ac towards enzyme degradation (Figure 3A). Due to the sensitivity of terminal Neu5Ac, enzymatic desialylation of GD1a gave GM1 (d18:1, C18:0) and GM1 (d20:1, C18:0), which were detected with *m*/*z* values of 1544.87 and 1572.90, respectively, in the scraped silica gel obtained from the GD1a area of TLC-separated gangliosides (Figure 3B; see Table 1, where the newly generated GM1 receptors are marked with red).These two lipoforms are the dominant degradation products of *V. cholerae* neuraminidase derived from the “precursors” GD1a (d18:1, C18:0) and GD1a (d20:1, C18:0) detected as remnant deprotonated [M−2H+Na]^−^ sodium adducts at *m*/*z* 1857.95 and 1885.99, respectively, and accompanied by multiple deprotonated sodium adducts marked with red numbers and explained in the spectrum (Figure 3B). The same GM1 species were detected after neuraminidase treatment in the silica gel extracts obtained from the TLC spots of the GD1b position, namely GM1 (d18:1, C18:0) and GM1 (d20:1, C18:0) with *m*/*z* values of 1544.85 and 1572.90, respectively (Figure 3C; for listing see Table 1). These species are descendants from GD1b (d18:1, C18:0) and GD1b (d20:1, C18:0), representing the remnant structures after enzyme treatment that appear as deprotonated [M−2H+Na]^−^ sodium adducts at *m*/*z* 1857.95 and 1885.99 in the spectrum as major ions together with GD1b (d20:1, C18:0) at *m*/*z* 1907.96. Their singly and multiply deprotonated and sodiated counterparts are detectable as minor ion signals and are assigned with red numbers, which are explained in the spectrum of Figure 3C. Collectively, the proof of principle of TLC overlay detection of GM1 molecules with choleragenoid confirmed the various lipoforms of primordial GM1 species from human brain ganglioside references and the additional GM1 receptors received by neuraminidase of *V. cholerae* showing its capability to generate additional GM1 receptors through conversion of the disialogangliosides GD1a and GD1b to GM1. Primordial GT1b and GT1b-derived GM1 (see Figure 2) were undetectable by MS analysis in the scraped silica samples from TLC plates most likely due to their low content.

### 3.4. TLC Overlay Detection of Choleragenoid-Binding GM1 in Ganglioside Preparations from pHCoEpiCs

**Figure 4:**
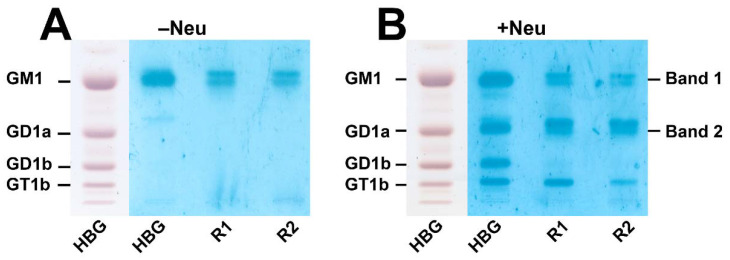
Detection of GM1 in two ganglioside preparations of pHCoEpiCs before (**A**) and after treatment with neuraminidase of *Vibrio cholerae* (**B**). GM1 was detected without (−Neu) and after neuraminidase treatment (+Neu) by means of the TLC overlay assay using choleragenoid (cholera toxin B subunit). Amounts of 15 µg of human brain gangliosides (HBG) were applied for the orcinol stains (left lanes of panel A and B). For the TLC overlay assays without (−Neu) and after neuraminidase treatment (+Neu) 0.2 µg of HBG and ganglioside amounts of the two replicates R1 and R2 of pHCoEpiCs, each corresponding to 2 × 10^5^ cells, were applied. HBG served as positive control for the TLC overlay assay. Structures of GM1, GD1a, GD1b and GT1b are depicted in Figure 1. Mass spectrometric characterized gangliosides obtained from the silica gel extracts of TLC bands at the positions of GM1 and GD1a (see Figure 5), assigned as band 1 and band 2, respectively, are listed in Table 2.

Figure 4 shows choleragenoid-mediated TLC overlay detection of GM1 in a TLC-separated preparation of gangliosides from pHCoEpiCs without neuraminidase (−Neu) treatment (Figure 4A) and after incubation of the chromatogram with the neuraminidase of *V. cholerae* (+Neu) (Figure 4B). The mixture of human brain gangliosides (HBG) served as positive control and the positions of GM1, GD1a, GD1b and GT1b are marked in the chromatograms. Both replicates (R1 and R2) from pHCoEpiCs showed positive reaction of choleragenoid at the position of GM1 in the TLC runs after incubation with the buffer control (−Neu) (Figure 4A). Further choleragenoid-recognized bands were detected in the two chromatograms of pHCoEpiC gangliosides after exposure to *V. cholerae* neuraminidase in the area of GD1a and GT1b (Figure 4B), indicating the generation of further GM1 receptors for the choleragenoid due to enzymatic desialylation of GD1a and GT1b serving as “precursors” of the detected GM1 species. Scraped silica gel from the positions of GM1 (Band 1) and GD1a (Band 2) were used for MS analysis as shown in the following paragraph. Primordial GT1b and GT1b-derived GM1 from the positions of GT1b in the chromatogram were undetectable by MS analysis in the scraped silica samples from TLC plates, most likely due to their low content.

### 3.5. Mass Spectrometric Characterization of Choleragenoid-Binding GM1 Lipoforms in Ganglioside Preparations of pHCoEpiCs

**Figure 5:**
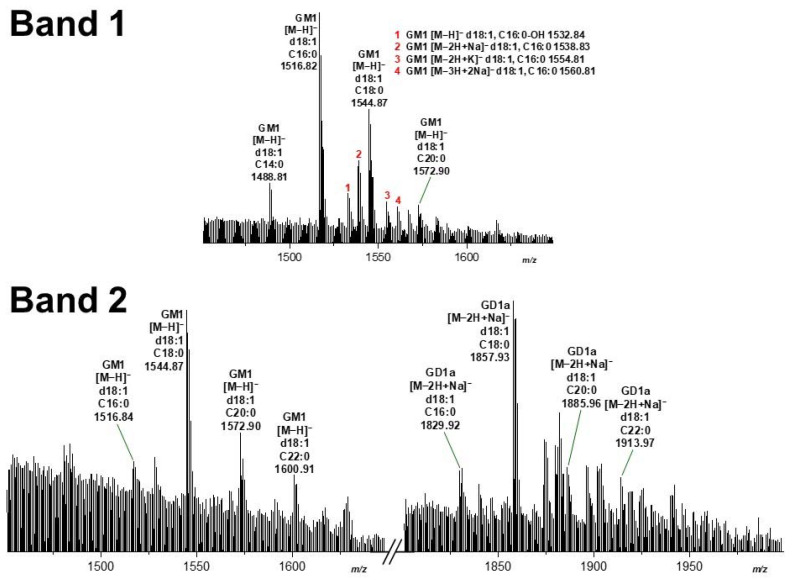
MS^1^ spectra of choleragenoid-detected GM1 species of pHCoEpiCs at TLC positions of GM1 (Band 1) and GD1a (Band 2) after treatment with neuraminidase of *Vibrio cholerae*. Separated gangliosides were extracted after *V. cholerae* neuraminidase treatment from scraped silica gel at positions of TLC-separated GM1 and GD1a, assigned as Band 1 and Band 2, respectively, in Figure 4B. Spectra were recorded in negative ion mode. The major signals of the deprotonated [M−H]^−^ species of GM1 and the deprotonated sodium adducts [M−2H+Na]^−^ of GD1a are listed in Table 2. Numbered minor species of GM1 (Band 1) are depicted on the right side of the panel.

Silica gel samples were prepared from two independent replicates of pHCoEpiCs, R1 and R2 (see Figure 4), from several TLC runs, and the extracts of band 1 and band 2 after exposure to *V. cholerae* neuraminidase (see Figure 4B) were combined, respectively, and submitted to mass spectrometric analysis. The MS^1^ spectra obtained from band 1 and band 2 using the negative ion mode are shown in the upper and lower part of Figure 5, respectively. Major primordial GM1 lipoforms from the TLC-separated band 1 were identified as GM1 (d18:1, C16:0) and GM1 (d18:1, C18:0) at *m*/*z* 1516.82 and 1544.87, respectively, detected as deprotonated species [M−H]^−^ as shown in the upper part (band 1) in Figure 5 and listed in Table 2. Minor GM1 lipoforms appearing as [M−H]^−^ were GM1 (d18:1, C14:0), GM1 (d18:1, C20:0) and hydroxylated GM1 (d18:1, C16:0-OH) at *m*/*z* 1488.81, 1572.90, and 1532.84, respectively. Further minor GM1 species were detected as deprotonated mono- and disodiated as well as kalium adducts of the GM1 (d18:1, C16:0) lipoform, marked with red numbers and explained in the upper right corner of band 1 in Figure 5. Common to all is the constant sphingosine (d18:1) moiety and variably saturated fatty acyl chains in the ceramide portion ranging from C14:0 up to C20:0.

Pretreatment of the TLC separated gangliosides of pHCoEpiCs resulted in GM1 (d18:1, C18:0) at *m*/*z* 1544.87 which exhibited the dominant ion signals obtained from the scraped silica gel of band 2 separating in the GD1a area in the chromatogram (band 2, left side in Figure 5). This strongest signal was accompanied by further minor signals to which the following structures could be assigned: GM1 (d18:1, C16:0) at *m*/*z* 1516.84, GM1 (d18:1, C20:0) at *m*/*z* 1572.90 and GM1 (d18:1, C22:0) at *m*/*z* 1600.91 (for listing see Table 2, where the newly generated GM1 receptors are marked with red). The remaining GD1a “precursors” after enzyme treatment with the corresponding lipid anchors Cer (d18:1, C16:0), Cer (d18:1, C18:0), Cer (d18:1, C20:0), and Cer (d18:1, C22:0) occurred as deprotonated sodium adducts in the spectrum at *m*/*z* 1829.92, 1857.93, 1885.96, and 1913.97, respectively, with GD1a (d18:1, C18:0) as the dominant species (band 2, right side in Figure 5 and Table 2). Common to all GD1a and GD1a-derived GM1 species are the constant sphingosine (d18:1) moiety and variably saturated fatty acyl chains in the ceramide portion ranging from C14:0 up to C22:0 as observed for primordial GM1, with the exception of the C22:0 lipoform detected in band 1. In summary, it remains to be noted that the neuraminidase of *V. cholerae* generated from the ganglioside fraction of pHCoEpiCs a set of further GM1 receptors for CT comprising descendants of the four “precursor” structures of the GD1a lipoforms with increasing fatty acyl chain lengths from C16:0 up to C22:0.

## 4. Discussion

Here we showed the recruitment of further GM1 receptors for choleragenoid by *V. cholerae* neuraminidase in addition to primordial cellular GM1 molecules through cleavage of the terminally linked Neu5Ac molecule of the disialoganglioside GD1a (see Figure 1), present in the ganglioside fraction of primary human colon epithelial cells (pHCoEpiCs), to the monosialoganglioside GM1. Serum-free conditions were chosen for cell cultivation because fetal calf serum, the most common serum supplement used in cell culture technology, has been shown in a study to work to contain the ganglio-series gangliosides GM1, GD1a, GD1b, and GT1b [26]. Exogenous gangliosides can be taken up by cultured cells and incorporated in the plasma membrane of in vitro propagated cells [29,30,31]. Therefore, the identified primordial GM1 and GD1a-derived choleragenoid-binding GM1 species recruited by *V. cholerae* neuraminidase can be considered as true endogenous gangliosides of colon epithelial cells. We can therefore rule out any doubt about their presence in pHCoEpiCs, because artificial incorporation of exogenous GM1, GD1a and other GSLs can be excluded due to the omission of the serum supplement. We provided data on the exact structures of the identified gangliosides of pHCoEpiCs employing preparative TLC overlay binding assays using choleragenoid combined with electrospray ionization mass spectrometry. The mixture of human brain gangliosides (HBG) served as a reference and positive control for reliability of in situ detection of the disialoganglioside GD1a and GD1a-derived GM1 as well as the original GM1 ganglioside in the silica gel samples scraped from the TLC plate. Furthermore, we considered the isomeric disialoganglioside GD1b of HBG as an additional control for the operating principle just as a proof of concept of the methodology as marked with arrows in Figure 2A for treatment of TLC-separated HBG with *V. cholerae* neuraminidase. GD1a is the reference ganglioside of interest because it represents the receptor-relevant ganglio-series disialoganglioside of pHCoEpiCs, which do express GD1a but no GD1b. The GM1 lipoforms and the higher sialylated gangliosides such as GD1a and GD1b of HBG, which served as a source for the enzymatic degradation to GM1, harbored Cer (d18:1, C18:0) and Cer (d20:1, C18:0) as lipid anchors. The long-chain sphingoid base C18-sphingosine (d18:1) is the main component of all sphingolipids and is always present in cell gangliosides [32,33,34], but only gangliosides of the brain and other human tissues of the central nervous system contain significant amounts of C20-sphingosine (d20:1) [35,36]. To be more precise, the four dominant gangliosides in the mammalian brain, namely GM1, GD1a, GD1b and GT1b, are quantitatively dominated by a single fatty acid (C18, stearic acid) attached to one of two sphingosines, 2-amino-4-octadecene-1,3-diol (d18:1) and 2-amino-4-dodecene-1,3-diol (d20:1) [37,38]. The gangliosides we detected in our proof of concept were GM1 (d18:1, C18:0) and GM1 (d20:1, C18:0), and the disialoganglioside GD1a (d18:1, C18:0) and GD1a (d20:1, C18:0) as well as GD1b (d18:1, C18:0) and GD1b (d20:1, C18:0). The ganglioside lipoforms with C20-sphingosine (d20:1) dominated somewhat over those with C18-sphingosine (d18:1), as suggested from the ion signal intensities in the respective mass spectra. Thus, the detected structures were in perfect agreement with the ones known to be present in mammalian nervous tissues including brain and neuronal cell cultures [35]. Importantly, no asialo-GM1 was detected in the neuraminidase-exposed GM1 band, thereby confirming the resistance of internal Neu5Ac at galatcose in position II of the Gg4Cer tetrahexosylceramide. A minor restriction of direct analysis of gangliosides in scraped silica gel samples might be missing low abundant ganglioside species with other ceramide structures than the detected ones, perhaps due to disturbing silica gel-derived compounds.

We endeavored to scrutinize the various lipoforms of the original GM1 ganglioside and GD1a-derived GM1 after neuraminidase treatment in the ganglioside preparations of pHCoEpiCs. The detected GM1 lipoforms and those of the “precursor” disialoganglioside GD1a of pHCoEpiCs were different when compared to those known from human neuronal tissues and neuronal cell cultures as mentioned above [35,37]. More precisely, both GM1 and GD1a were characterized by invariable C18-sphingosine (d18:1), and a panel of variable saturated fatty acyl chain length of C14:0, C16:0; C18:0, C20:0 and, in the case of GD1a, also of C22:0. Interestingly, no very long-chain C24 fatty acyl chains, either saturated nor unsaturated, were found in the ceramides of GM1 and GD1a of pHCoEpiCs. An alternative MS analysis of total gangliosides might be useful in detecting minor gangliosides, if present, due to higher sensitivity. This might be a limitation of preparative TLC combined with MS analysis as done in this study when compared to MS analysis of total gangliosides. However, neuraminidase treatment of total gangliosides and the ensuing allocation of GM1 to higher sialylated “precursor” gangliosides such as GD1a or GD1b, where GM1 species might derive from, is problematic. This is the special advantage of in situ detection of GM1 directly at the position of GD1a on the TLC plate as was done for pHCoEpiCs here. This modus operandi allows for the unambiguous assignment of GM1 to GD1a as the ganglioside of origin.

In the context of other ganglioside-binding toxins, it might be of interest that the heat-labile (LT) enterotoxin LT-IIc of *E. coli* was found to bind to gangliosides with Neu5Acα2-3Galβ1-3GalNAc terminus such as GM1 with key distinctions in specificity [39]. Interestingly, LT-IIc did not bind to GM1 with short-chain (C16) fatty acyl ceramides but recognized the GM1 ganglioside with long-chain (C24) fatty acyl ceramide. Thus, the binding of the ganglioside-specific LT-IIc toxin is not just dependent on carbohydrate composition, but also on the orientation of the glycan portion of GM1 by the ceramide moiety suggesting the contribution of long-chain fatty acyl ceramides to host cell interactions [39]. A similar binding specificity towards GM1 has been described for simian virus 40 (SV40). The interaction of SV40 with GM1 induced membrane curvature that proceeded to the formation of invaginations and tubules in the plasma membrane as well as in giant unilamellar vesicles. However, only GM1 molecules with long acyl chains effected these tubular structures, whereas GM1 with short hydrocarbon chains failed to raise such invagination [40].

The interaction of CT with GM1 in lipid rafts of the apical membrane of epithelial cells and the intracellular trafficking of CT depends largely on the GM1 acyl chains rather than the carbohydrate head groups. This has been shown in early studies with human colon T84 and Caco 2 cell lines, suggesting that the CT-mediated signal transduction depends on binding and/or clustering, specifically the ganglioside GM1, and that the ganglioside structure dictates toxin action [41]. Therefore, the association of GM1 in clusters such as lipid rafts explains how GM1 molecules can function as sorting motifs for CT to move the toxin backwards from the plasma membrane through endosomes and the *trans*-Golgi network (TGN) to the ER [9]. Recently, it was been shown that structured clustering of GM1 is required for membrane curvature induced by CT, a mechanism that requires both the multiplicity and specific geometry of GM1 binding sites for the induction of membrane curvature and thus for the toxin‘s entry into host cells [42].

Previous studies have shown that different subcellular lipid sorting is determined by the GM1 ceramide acyl chain. Using the human A431 epithelial cell line, only GM1 with unsaturated acyl chains was found to sort efficiently from the plasma membrane to the TGN and ER, exemplarily shown for the GM1-C16:0 and GM1-C16:1 pair, whereas GM1-C18:0 with saturated fatty acid entered the ER very inefficiently [43]. Moreover, CT has been observed to intracellularly move from the endosomes across the cell to the basolateral plasma membrane in polarized epithelial cells by transcytosis, thus overcoming the intestinal barrier [13]. Specific sorting of a fraction of CT into this transcytotic pathway bypasses the retrograde route to the TGN mediated by GM1-lipoforms with *cis*-unsaturated or short acyl chains in the ceramide moiety, suggesting that the structure of the ceramide domain dictates the various possible retrograde pathways of CT [13]. These data suggest that differential sorting of various GM1 lipoforms may explain the ability of CT to enter host cells by different endocytic pathways and to direct the CT retrogradely into multiple subcellular compartments. These findings open up questions regarding the functional impact of those GM1 lipoforms detected by us in pHCoEpiCs. GM1 lipoforms were found to harbor unexceptionally saturated fatty acyl chains, with C16:0 and C18:0 structures as the major and C14:0 and C20:0 as the minor ones. However, elucidating the meaning of this structural homology of saturated fatty acyl chains and variability in chain lengths of the GM1 lipoforms of pHCoEpiCs remains a task to be solved for the future. Regardless, the observed ceramide heterogeneity of GM1 species from various colon cell types may provide a molecular explanation for the specificity and diversity of the retrograde transport of CT in host cells.

All of these experimental approaches underscore the importance of precise structural analysis of both the glycan portion and the hydrophobic lipid moiety of GSLs as well. In the past, the biological impact of ceramide has been underestimated just to function as a lipid anchor and to hold the molecules in the outer leaflet of the plasma membrane. This view has changed and we are now proceeding in identifying the multiple functional roles of the GSLs’ heterogeneity of the hydrophobic moiety in membrane biology in general and especially in cellular interaction processes of GSLs interacting with pathogens such as viruses and bacteria or bacterial toxins. Collectively, the functional role of choleragenoid-binding GM1 lipoforms in primary human epithelial cells of the intestine regarding the initial interaction of the toxin with the plasma membrane and the ensuing internalization and retrograde trafficking of the CT-GM1-complex to the intracellular targets is now (as before) largely uncertain and remains to be explored in future studies.

## 5. Conclusions

So far, almost nothing was known about the ganglioside profile of primary epithelial cells from the human colon regarding the GM1 lipoforms and those GM1 species which could be generated from higher sialylated gangliosides with a GM1 core structure by the neuraminidase from pathogenic *V. cholerae*. We have now closed this knowledge gap with this article by providing the structures of the various lipoforms of primordial GM1 and the GM1 species derived from neuraminidase-desialylated GD1a. Collectively, the resistance of the internal Neu5Ac of higher sialylated GD1a and GT1b detected in the ganglioside fraction of pHCoEpiCs allowed the bacterial neuraminidase to generate further cholera toxin receptors from these gangliosides. The specific action of *V. cholerae* neuraminidase recruiting further toxin receptors from its own cellular “precursor” di- and trisialogangliosides of the colon epithelium might be of advantage for the pathogen’s propagation and persistence in the human gut or spreading in the environment. However, our findings cannot be generalized for other cell types, because only one batch of primary epithelial cells from human colon has yet to be analyzed. Groundbreaking advancements achieved with newly developed secondary matrix-assisted laser desorption/ionization mass spectrometry (MALDI MS) imaging technologies allows for label-free in situ detection of glycolipids in tissue or organ sections as well as cell cultures at subcellular resolution [44,45,46]. Moreover, MS imaging can simultaneously record the lateral distribution of numerous biomolecules in tissue slices, and provides precise structural details of membrane constituents on the cellular niveau with high resolution in mass and space at the single-cell level [47]. This technology combined with the cholera toxin as a probe for membrane biology offers an innovative model for exploring membrane structure and the dynamics of nanodomain assembly [48]. Furthermore, glycolipid-based applications of MS imaging may have the power of a diagnostic tool for resolving lipid-related pathological conditions; for instance, in human colon epithelium. A further aspect of MS research in the future is directed to developing three-dimensional MS imaging with the aim to study the topographic distribution of compounds exposed on irregular 3D surfaces with single-cell metabolomics profiling and operating with subcellular resolution [49].

## Figures and Tables

**Table 1:** GM1 and GD1a- and GD1b-derived GM1 species of human brain gangliosides obtained by *V. cholerae* neuraminidase treatment ^1^.

TLC Band	Ceramide	Formula	*m*/*z*_exp_^4^	*m*/*z*_calc_^4^
GM1 band ^2^				
GM1	d18:1, C18:0	II^3^Neu5Ac-Gg4Cer	1544.87	1544.8688
GM1	d20:1, C18:0	II^3^Neu5Ac-Gg4Cer	1572.90	1572.9001
GD1a band ^3^				
GM1	d18:1, C18:0	II^3^Neu5Ac-Gg4Cer	1544.87	1544.8688
GM1	d20:1, C18:0	II^3^Neu5Ac-Gg4Cer	1572.90	1572.9001
GD1a	d18:1, C18:0	IV^3^Neu5Ac, II^3^Neu5Ac-Gg4Cer	1857.95	1857.9462
GD1a	d20:1, C18:0	IV^3^Neu5Ac, II^3^Neu5Ac-Gg4Cer	1885.99	1885.9775
GD1b band ^3^				
GM1	d18:1, C18:0	II^3^Neu5Ac-Gg4Cer	1544.85	1544.8688
GM1	d20:1, C18:0	II^3^Neu5Ac-Gg4Cer	1572.90	1572.9001
GD1b	d18:1, C18:0	II^3^(Neu5Ac)_2_-Gg4Cer	1857.95	1857.9462
GD1b	d20:1, C18:0	II^3^(Neu5Ac)_2_-Gg4Cer	1885.99	1885.9775
GD1b	d20:1, C18:0	II^3^(Neu5Ac)_2_-Gg4Cer	1907.96	1907.9594

^1^ GM1, GD1a and GD1b species were detected by mass spectrometry in the negative ion mode in a human brain ganglioside (HBG) preparation (see Figure 3). Ions were obtained from the scraped silica gel of the GM1 band, the GD1a band and the GD1b band of choleragenoid-positive TLC-separated HBG (see Figure 2). ^2^ The GM1 band (Figure 2A) contains TLC overlay-detected GM1 using choleragenoid without *V. cholerae* neuraminidase treatment (−Neu). ^3^ The GD1a band (Figure 2B) and the GD1b-band (Figure 2C) contain TLC overlay-detected GM1 derived from GD1a or GD1b, respectively, using choleragenoid after *V. cholera* neuraminidase treatment (+Neu) and some remaining intact GD1a or GD1b, respectively, after enzyme treatment. Only the deprotonated [M−H]^−^ species of GM1 and the deprotonated sodium adducts [M−2H+Na]^−^ of GD1a and the [M−2H+Na]^−^ and [M−3H+2Na]^−^ adducts of GD1b representing the major signals are listed (see Figure 3). Newly recruited additional cholera toxin receptors by neuraminidase treatment are marked in red. ^4^ exp, experimental; calc, calculated.

**Table 2:** Proposed structures of GM1 and GD1a-derived GM1 species of pHCoEpiCs obtained by *V. cholerae* neuraminidase treatment ^1^.

TLC Band	Ceramide	Formula	*m*/*z*_exp_^4^	*m*/*z*_calc_^4^
Band 1 ^2^				
GM1	d18:1, C14:0	II^3^Neu5Ac-Gg4Cer	1488.81	1488.8062
GM1	d18:1, C16:0	II^3^Neu5Ac-Gg4Cer	1516.82	1516.8375
GM1	d18:1, C16:0-OH	II^3^Neu5Ac-Gg4Cer	1532.84	1532.8324
GM1	d18:1, C18:0	II^3^Neu5Ac-Gg4Cer	1544.87	1544.8688
GM1	d18:1, C20:0	II^3^Neu5Ac-Gg4Cer	1572.90	1572.9001
Band 2 ^3^				
GM1	d18:1, C16:0	II^3^Neu5Ac-Gg4Cer	1516.84	1516.8375
GM1	d18:1, C18:0	II^3^Neu5Ac-Gg4Cer	1544.87	1544.8688
GM1	d18:1, C20:0	II^3^Neu5Ac-Gg4Cer	1572.90	1572.9001
GM1	d18:1, C22:0	II^3^Neu5Ac-Gg4Cer	1600.91	1600.9314
GD1a	d18:1, C16:0	IV^3^Neu5Ac, II^3^Neu5Ac-Gg4Cer	1829.92	1829.9149
GD1a	d18:1, C18:0	IV^3^Neu5Ac, II^3^Neu5Ac-Gg4Cer	1857.93	1857.9462
GD1a	d18:1, C20:0	IV^3^Neu5Ac, II^3^Neu5Ac-Gg4Cer	1885.96	1885.9775
GD1a	d18:1, C22:0	IV^3^Neu5Ac, II^3^Neu5Ac-Gg4Cer	1913.97	1914.0088

^1^ GM1 and GD1a species were detected by mass spectrometry in negative ion mode (see Figure 5) in extracts obtained from a ganglioside preparation of pHCoEpiCs. Ions were obtained from scraped silica gel of band 1 (GM1) and band 2 (GD1a and GD1a-derived GM1) of choleragenoid-positive TLC-separated gangliosides from pHCoEpiCs (see Figure 4). ^2^ Band 1 (Figure 4A) contains TLC overlay-detected GM1 using choleragenoid without *V. cholerae* neuraminidase treatment. ^3^ Band 2 (Figure 4B) contains TLC overlay-detected GM1 derived from GD1a using choleragenoid after *V. cholerae* neuraminidase treatment and some remaining intact GD1a after enzyme treatment. Only deprotonated [M−H]^−^ species of GM1 are listed, while disialylated GD1a species appear as deprotonated sodium adducts [M−2H+Na]^−^ as listed (see Figure 5). Newly recruited additional cholera toxin receptors by neuraminidase treatment are marked in red. ^4^ exp, experimental; calc, calculated.

## Data Availability

Data produced throughout the study is available from the corresponding author upon reasonable request.

## References

[1] Mandal S., Mandal M.D., Pal N.K. (2011). Cholera: A great global concern. Asian Pac. Trop. Med..

[2] Harris J.B., LaRocque R., Qadri F., Ryan E.T., Calderwood S.B. (2012). Cholera. Lancet.

[3] Ramamurthy T., Nandy R.K., Mukhopadhyay A.K., Dutta S., Mutreja A., Okamoto K., Miyoshi S.I., Nair G.B., Ghosh A. (2020). Virulence regulation and innate host response in the pathogenicity of *Vibrio cholerae*. Front. Cell. Infect. Microbiol..

[4] Baldauf K.J., Royal J.M., Hamorsky K.T., Matoba N. (2015). Cholera toxin B: One subunit with many pharmaceutical applications. Toxins.

[5] Spangler B.D. (1992). Structure and function of cholera toxin and the related *Escherichia coli* heat-labile enterotoxin. Microbiol. Rev..

[6] Zhang R.G., Westbrook M.L., Westbrook E.M., Scott D.L., Otwinowski Z., Maulik P.R., Reed R.A., Shipley G.G. (1995). The 2.4 Å crystal structure of cholera toxin B subunit pentamer: Choleragenoid. J. Mol. Biol..

[7] Zhang R.G., Scott D.L., Westbrook M.L., Nance S., Spangler B.D., Shipley G.G., Westbrook E.M. (1995). The three-dimensional crystal structure of cholera toxin. J. Mol. Biol..

[8] Lencer W.I., Saslowsky D. (2005). Raft trafficking of AB_5_ subunit bacterial toxins. Biochim. Biophys. Acta.

[9] Chinnapen D.J., Chinnapen H., Saslowsky D., Lencer W.I. (2007). Rafting with cholera toxin: Endocytosis and trafficking from plasma membrane to ER. FEMS Microbiol. Lett..

[10] Wernick N.L., Chinnapen D.J., Cho J.A., Lencer W.I. (2010). Cholera toxin: An intracellular journey into the cytosol by way of the endoplasmic reticuluim. Toxins.

[11] Lencer W.I., Tsai B. (2003). The intracellular voyage of cholera toxin: Going retro. Trends Biochem. Sci..

[12] Sánchez J., Holmgren J. (2008). Cholera toxin structure, gene regulation and pathophysiological and immunological aspects. Cell. Mol. Life Sci..

[13] Saslowsky D.E., te Welscher Y.M., Chinnapen D.J., Wagner J.S., Wan J., Kern E., Lencer W.I. (2013). Ganglioside GM1-mediated transcytosis of cholera toxin bypasses the retrograde pathway and depends on the structure of the ceramide domain. J. Biol. Chem..

[14] Kuhn R., Wiegandt H. (1963). Die Konstitution der Ganglio-*N*-tetraose und des Gangliosids G_I_. Chem. Ber..

[15] Chester M.A. (1998). IUPAC-IUB Joint Commission on Biochemical Nomenclature (JCBN). Nomenclature of glycolipids—recommendations 1997. Eur. J. Biochem..

[16] Aureli M., Mauri L., Ciampa M.G., Prinetti A., Toffano G., Secchieri C., Sonnino S. (2016). GM1 ganglioside: Past studies and future potential. Mol. Neurobiol..

[17] Svennerholm S. (1963). Chromatographic separation of human brain gangliosides. J. Neurochem..

[18] Svennerholm L. (1964). The gangliosides. J. Lipid Res..

[19] Holmgren J., Lönnroth I., Svennerholm L. (1973). Tissue receptor for cholera exotoxin: Postulated structure from studies with GM1 ganglioside and related glycolipids. Infect. Immun..

[20] Fishman P.H. (1982). Role of membrane gangliosides in the binding and action of bacterial toxins. J. Membr. Biol..

[21] Holmgren J., Lönnroth I., Månsson J.E., Svennerholm L. (1975). Interaction of cholera toxin and membrane GM1 ganglioside of small intestine. Proc. Natl. Acad. Sci. USA.

[22] Detzner J., Krojnewski E., Pohlentz G., Steil D., Humpf H.U., Mellmann A., Karch H., Müthing J. (2021). Shiga toxin (Stx)-binding glycosphingolipids of primary human renal cortical epithelial cells (pHRCEpiCs) and Stx-mediated cytotoxicity. Toxins.

[23] Detzner J., Klein A.L., Pohlentz G., Krojnewski E., Humpf H.U., Mellmann A., Karch H., Müthing J. (2021). Primary human renal proximal tubular epithelial cells (pHRPTEpiCs): Shiga toxin (Stx) glycosphingolipid receptors, Stx susceptibility, and interaction with membrane microdomains. Toxins.

[24] Detzner J., Püttmann C., Pohlentz G., Humpf H.-U., Mellmann A., Karch H., Müthing J. (2021). Primary human colon epithelial cells (pHCoEpiCs) do express the Shiga toxin (Stx) receptor glycosphingolipids Gb3Cer and Gb4Cer and are largely refractory but not resistant towards Stx. Int. J. Mol. Sci..

[25] Müthing J., Egge H., Kniep B., Mühlradt P.F. (1987). Structural characterization of gangliosides from murine T lymphocytes. Eur. J. Biochem..

[26] Müthing J., Pörtner A., Jäger V. (1992). Ganglioside alterations in YAC-1 cells cultivated in serum-supplemented and serum-free growth medium. Glycoconj. J..

[27] Müthing J. (1996). High-resolution thin-layer chromatography of gangliosides. J. Chromatogr. A.

[28] Detzner J., Pohlentz G., Müthing J. (2021). Thin-layer chromatography in structure and recognition studies of Shiga toxin glycosphingolipid receptors. Methods Mol. Biol..

[29] Radsak K., Schwarzmann G., Wiegandt H. (1982). Studies on the cell association of exogenously added sialo-glycolipids. Hoppe Seylers Z. Physiol. Chem..

[30] Saqr H.E., Pearl D.K., Yates A.J. (1993). A review and predictive models of ganglioside uptake by biological membranes. J. Neurochem..

[31] Schwarzmann G. (2001). Uptake and metabolism of exogenous glycosphingolipids by cultured cells. Semin. Cell Dev. Biol..

[32] Sonnino S., Mauri L., Chigorno V., Prinetti A. (2007). Gangliosides as components of lipid membrane domains. Glycobiology.

[33] Müthing J., Distler U. (2010). Advances on the compositional analysis of glycosphingolipids combining thin-layer chromatography with mass spectrometry. Mass Spectrom. Rev..

[34] Merrill A.H. (2011). Sphingolipid and glycosphingolipid metabolic pathways in the era of sphingolipidomics. Chem. Rev..

[35] Sonnino S., Chigorno V. (2000). Ganglioside molecular species containing C18- and C20-sphingosine in mammalian nervous tissues and neuronal cell cultures. Biochim. Biophys. Acta.

[36] Lunghi G., Fazzari M., Di Biase E., Mauri L., Chiricozzi E., Sonnino S. (2021). The structure of gangliosides hides a code for determining neuronal functions. FEBS Open Bio.

[37] Jackson S.N., Colsch B., Egan T., Lewis E.K., Schultz J.A., Woods A.S. (2011). Gangliosides’ analysis by MALDI-ion mobility MS. Analyst.

[38] Schnaar R.L. (2016). Gangliosides of the vertebrate nervous system. J. Mol. Biol..

[39] Berenson C.S., Nawar H.F., Kruzel R.L., Mandell L.M., Connell T.D. (2013). Ganglioside-binding specificities of *E. coli* enterotoxin LT-IIc: Importance of long-chain fatty acyl ceramide. Glycobiology.

[40] Ewers H., Römer W., Smith A.E., Bacia K., Dmitrieff S., Chai W., Mancini R., Kartenbeck J., Chambon V., Berland L. (2010). GM1 structure determines SV40-induced membrane invagination and infection. Nat. Cell Biol..

[41] Badizadegan K., Wolf A.A., Rodighiero C., Jobling M., Hirst T.R., Holmes R.K., Lencwer W.I. (2020). Floating cholera toxin into epithelial cells: Functional association with caveolae-like detergent-insoluble membrane microdomains. Int. J. Med. Microbiol..

[42] Kabbani A.M., Raghunathan K., Lencer W.I., Kenworthy A.K., Kelly C.V. (2020). Structured clustering of the glycosphingolipid GM1 is required for membrane curvature induced by cholera toxin. Proc. Natl. Acad. Sci. USA.

[43] Chinnapen D.J., Hsieh W.T., te Welscher Y.M., Saslowsky D., Kaoutzani L., Brandsma E., D’Auria L., Park H., Wagner J.S., Drake K.R. (2012). Lipid sorting by ceramide structure from plasma membrane to ER for the cholera toxin receptor ganglioside GM1. Dev. Cell.

[44] Soltwisch J., Kettling H., Vens-Cappell S., Wiegelmann M., Müthing J., Dreisewerd K. (2015). Mass spectrometry imaging with laser-induced postionization. Science.

[45] Niehaus M., Soltwisch J., Belov M.E., Dreisewerd K. (2019). Transmission-mode MALDI-2 mass spectrometry imaging of cells and tissues at subcellular resolution. Nat. Methods.

[46] Dreisewerd K., Bien T., Soltwisch J. (2022). MALDI-2 and t-MALDI-2 mass spectrometry. Methods Mol. Biol..

[47] Capolupo L., Khven I., Lederer A.R., Mazzeo L., Glousker G., Ho S., Russo F., Paz Montoya J., Bhandari D.R., Bowman A.P. (2022). Sphingolipids control dermal fibroblast heterogeneity. Science.

[48] Kennworthy A.K., Schmieder S.S., Raghunathan K., Tiwari A., Wang T., Kelly C.V., Lencer W.I. (2021). Cholera toxin as a probe for membrane biology. Toxins.

[49] Dreisewerd K., Yew J.Y. (2017). Mass spectrometry imaging goes three dimensional. Nat. Methods.

